# Expression and prognostic value of CD97 and its ligand CD55 in pancreatic cancer

**DOI:** 10.3892/ol.2014.2751

**Published:** 2014-12-01

**Authors:** ZHENG HE, HUI WU, YANLI JIAO, JUN ZHENG

**Affiliations:** Department of General Surgery, The First College of Clinical Medical Sciences, Three Gorges University, Yichang, Hubei 443002, P.R. China

**Keywords:** pancreatic cancer, CD97, CD55, prognosis, aggressiveness

## Abstract

CD97 is a member of the epidermal growth factor-seven transmembrane family. It affects tumor aggressiveness by binding its cellular ligand CD55 and exhibits adhesive properties. Previous studies have shown that CD97 and CD55 are involved in the dedifferentiation, migration, invasiveness and metastasis of tumors. However, little is known regarding the roles of CD97 and CD55 in pancreatic cancer. In this study, immunohistochemistry was used to analyze CD97 and CD55 protein expression in samples obtained from 37 pancreatic cancer patients. CD97 and CD55 were absent or only weakly expressed in the normal pancreatic tissues but strongly expressed in pancreatic cancer tissues (P<0.05), particularly in tissues with lymph node involvement, metastasis or vascular invasion (P<0.05). Notably, CD97 and CD55 were expressed consistently in pancreatic cancer tissues (*r*^2^=0.5422; P<0.05). In addition, CD97 and CD55 expression levels were found to significantly correlate with tumor aggressiveness (P<0.01). Multivariate analyses revealed that CD97 and CD55 expression levels were closely associated with prognosis (P<0.05). Taken together, these results indicated that CD97 and its ligand CD55 are upregulated in pancreatic cancers and are closely associated with lymph node involvement, metastasis and vascular invasion. Thus, analysis of both CD97 and CD55 expression may present potential prognostic value for pancreatic cancer.

## Introduction

Pancreatic cancer is the sixth most common cause of cancer-related mortality in China (1,2). Pancreatic cancer exhibits an extremely poor prognosis, possibly as it is often diagnosed at an advanced stage, despite recent advances in diagnostic methods. At present, no adjuvant chemotherapy regimens have been identified for the treatment of pancreatic cancer, due to problems regarding drug resistance and limited efficacy. Curative surgical approaches remain the standard treatment; however, the prognosis of pancreatic cancer remains poor with regard to postsurgical five-year survival rates (3). Thus, the identification of novel prognostic markers for pancreatic cancer is critical to assess invasion and metastasis and to aid with the management of postoperative treatment for high-risk patients.

CD97 is an important member of the epidermal growth factor-seven transmembrane family (4,5), which is associated with aggressiveness and lymph node involvement in thyroid tumors, colorectal cancer, oral squamous cell carcinomas and primary gallbladder carcinoma (6–9). Based on its expression pattern and structure, CD97 has been hypothesized to be involved in cellular adhesion via interactions with other cell-surface and extracellular matrix proteins (10). Through its epidermal growth factor domain region, CD97 binds to CD55, which affects cancer invasion and metastasis (6,11,12). CD55 is also termed complement decay accelerating factor, as it mediates the complement activation pathway (4,13). The complement immune system consists of a number of glycoproteins, which are involved in an enzymatic cascade, that upon activation results in the formation of the membrane attack complex that leads to cell lysis (4,7). A number of studies have demonstrated that CD97 and CD55 are involved in tumor dedifferentiation, migration, invasiveness and metastasis (14). However, the association between CD97 and CD55 expression in pancreatic cancer has not yet been systemically investigated. Therefore, in this study, CD97 and CD55 expression in pancreatic cancer tissues and adjacent normal pancreatic tissues was investigated with regard to tumor aggressiveness and prognosis.

## Materials and methods

### Patients and tissue samples

Surgical pancreatic cancer and adjacent normal pancreatic tissue specimens were obtained from 37 patients at The First College of Clinical Medical Sciences, Three Gorges University (Yichang, China) between January 2009 and December 2010. Written informed consent was obtained from all patients. A total of 18 male and 19 female patients were included, with a mean age of 56.7 years (range: 47–64 years). No patients had received preoperative radiotherapy or chemotherapy. Histologically, all 37 cancer specimens were stained using hematoxylin and eosin and were subsequently diagnosed as pancreatic adenocarcinomas. The tumor stage of the specimens was classified according to the American Joint Committee on Cancer staging manual (15). All protocols were approved by the institutional review board of The First College of Clinical Medical Sciences, Three Gorges University. The cancer specimens were graded as well-, moderately or poorly differentiated adenocarcinoma according to the National Comprehensive Cancer Network classification (16). The majority of the cancers (32/37) were moderately differentiated, four were well-differentiated and one was poorly differentiated. The 37 patients included in this study were followed up for three years. This study was approved by the Human Research Ethics Committees at The First College of Clinical Medical Sciences, Three Gorges University.

### Immunohistochemistry

Paraffin sections (3-μm) were incubated overnight at 4°C with primary antibodies against CD55 (1:50; goat polyclonal antibody; cat. no. sc-7067; Santa Cruz Biotechnology, Inc., Santa Cruz, CA, USA) and CD97 (1:100; rabbit polyclonal antibody; cat. no. sc-98577; Santa Cruz Biotechnology, Inc.), washed and incubated with biotinylated goat anti-rabbit IgG (1:50; goat anti-rabbit monoclonal antibody; cat. no. BA1003; Boster Biotechnology, Inc., Wuhan, China) for 30 min. Next, sections were washed three times with phosphate-buffered saline, stained with diaminobenzidine and examined by light microscopy (Olympus BX52; Olympus Corporation, Tokyo, Japan). Six randomly selected fields from each sample were examined by two pathologists, who were blinded to patient diagnosis and outcome. Areas positively expressing CD55 and CD97, and the average optical density were recorded and analyzed using Image-Pro Plus version 6.0 (Media Cybernetics, Inc., Rockville, MD, USA) software.

### Statistical analysis

Results are presented as the mean ± standard deviation. The treatment groups were compared using independent sample t-tests. One way analysis of variance was used for two-sided pair-wise multiple comparisons. P<0.05 was considered to indicate a statistically significant difference. All analyses were performed using SPSS software, version 17.0 (SPSS Inc., Chicago, IL, USA).

## Results

### Clinicopathological characteristics of CD97 expression in pancreatic cancer

**Figure 1:**
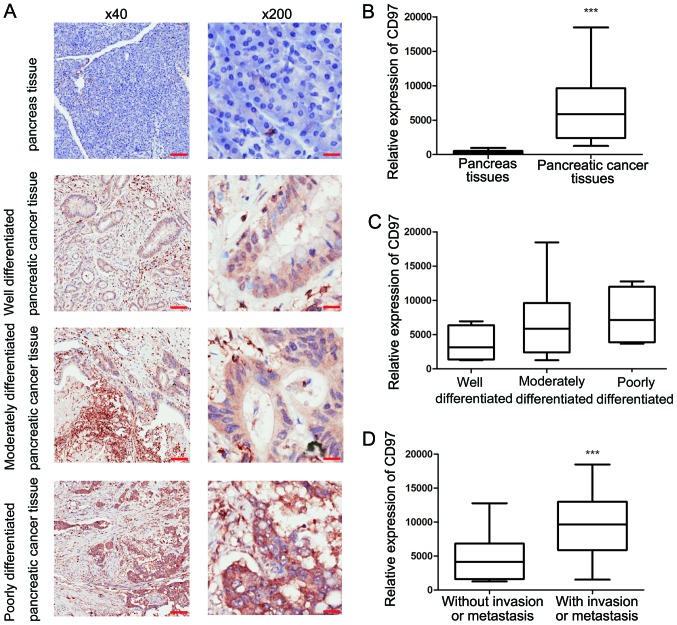
CD97 expression in normal pancreatic and pancreatic cancer tissues. (A) Immunohistochemical analysis of CD97 expression in normal pancreatic tissue and in well-, moderately and poorly differentiated pancreatic cancer tissues. Areas positive for CD97 expression and the average optical density (AOD) were recorded using Image-Pro Plus Version 6.0 software. Every sample was randomly analyzed from three, ×100 fields of vision. We then used the AOD values as an indication of the relative quantity of CD97, and quantitatively analyzed CD97 expression in the following three situations. (B) Quantitative analysis of CD97 expression in normal pancreatic tissue compared with pancreatic cancers. CD97 expression was higher in pancreatic cancers than in normal tissues (P<0.0001). (C) Quantitative analysis of CD97 expression in pancreatic cancers classified by grades of differentiation. There was no significant difference in the expression of CD97 between the differentiation grades. (D) Quantitative analysis of CD97 expression in tumors from patients without and with invasion and/or metastasis. CD97 expression was significantly higher in patients with invasion or metastasis than those without invasion or metastasis (P=0.0018).

CD97 expression in pancreatic cancer and adjacent normal pancreatic tissues was investigated. In adjacent normal pancreatic tissues, CD97 staining was absent or weakly positive and was significantly localized in the cytoplasm of tumor cells, particularly in tumors with lymph node involvement or distant metastases (Fig. 1A). All 37 pancreatic cancer tissue specimens were CD97^+^, including 15 tumors with extremely strong CD97^+^ staining and four tumors with uniform CD97 staining. All 15 tumors exhibiting high CD97 expression had lymph node involvement and of the four tumors with uniform CD97 staining, two exhibited vascular invasion and one had distant metastasis. Based on the Image-Pro Plus analysis results, CD97 expression in pancreatic cancer tissues was significantly higher than that of the adjacent normal pancreatic tissues (P<0.0001; Fig. 1B). Furthermore, tumors with lymph node involvement, vascular invasion or distant metastasis exhibited a significantly higher level of CD97, when compared with that of carcinomas without (P=0.0018; Fig. 1D). Notably, no significant difference was identified between CD97 expression and clinical parameters, including age, gender and differentiation (Fig. 1C).

### Clinicopathological characteristics of CD55 expression in pancreatic cancer

**Figure 2:**
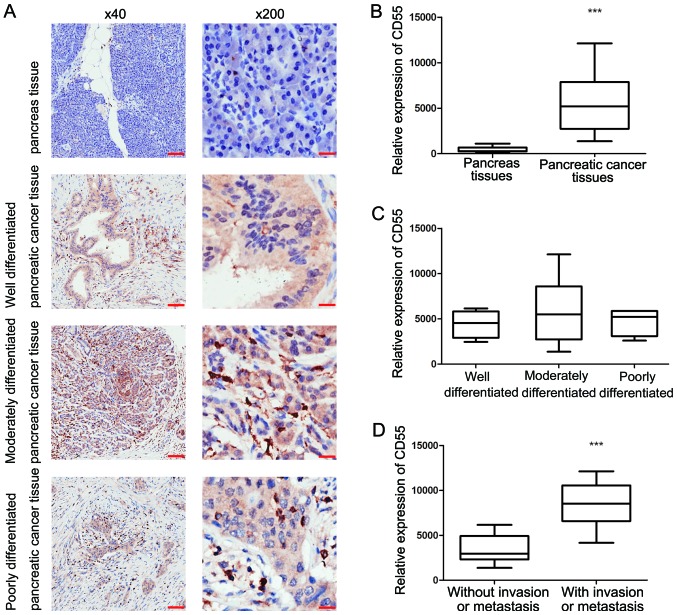
CD55 expression in normal pancreatic and pancreatic cancer tissues. (A) Immunohistochemical analysis of CD55 expression in normal pancreatic tissue and in well-, moderately and poorly differentiated pancreatic cancer tissues. (B) Quantitative analysis of CD55 expression in normal pancreatic tissue, and pancreatic cancer tissue revealed that CD55 expression was higher in pancreatic cancer tissues when compared with that in normal pancreas tissues (P<0.0001). (C) Quantitative analysis of CD55 expression in pancreatic cancers classified by grades of differentiation. There was no significant difference in the expression of CD55 between the differentiation grades. (D) Quantitative analysis of CD55 expression in tumors from patients without and with invasion and/or metastasis. CD55 expression was significantly higher in patients with invasion or metastasis than those without invasion or metastasis (P<0.0001).

The expression of CD55 in pancreatic cancer and adjacent normal pancreatic tissues was investigated. CD55 staining was absent or weakly positive in normal pancreatic tissues. However, strong CD55^+^ expression was exhibited in the cell membranes in pancreatic cancer tissues (Fig. 2A). All 37 pancreatic cancer tissue specimens were CD55^+^, including 13 tumors with strong CD55 expression and four tumors with uniform CD55^+^ staining. All 13 CD55^+^ tumors had lymph node involvement, and of the four tumors with uniform CD55^+^ staining, two exhibited vascular invasion and one exhibited distant metastasis. Based on the Image-Pro Plus analysis results, CD97 expression levels in pancreatic cancer tissues are significantly higher than that of adjacent normal pancreatic tissues (P<0.0001; Fig. 2B). In tumors with lymph node involvement, vascular invasion or distant metastasis, carcinomas exhibited a significantly higher level of CD97^+^ expression when compared with carcinomas without (P<0.0001; Fig. 2D). Notably, no significant differences between clinical parameters, including age, gender and differentiation, and CD97 expression were identified (Fig. 2C).

### Correlation between CD97 and CD55 expression and tumor aggressiveness and prognosis

**Figure 3:**
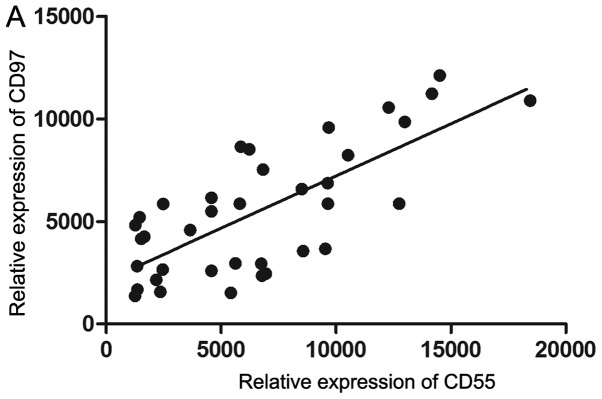

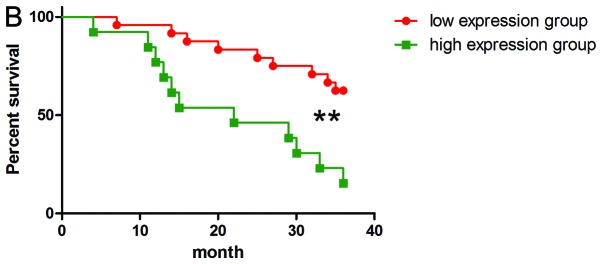
Correlation between CD97 and CD55 expression and tumor aggressiveness and prognosis. (A) The expression pattern of CD97 was found to significantly correlate with that of CD55 (r^2^=0.5422, P<0.0001). (B) Kaplan-Meier plots revealed significantly poorer survival rate at all time points for the high CD97 and CD55 expression group compared with the low CD97 and CD55 expression group (P<0.01).

The expression of CD97 was found to significantly correlate with CD55 expression (*r*^2^=0.5422; P<0.0001; Fig. 3A). A total of thirteen patients had a significantly stronger expression of CD97 and CD55 than the other 24 patients. These 13 patients were defined as the CD97/CD55^high^ expression group and the remaining 24 patients were the CD97/CD55^low^ expression group. The co-expression of CD97 and CD55 was associated with lymph node involvement, metastasis and vascular invasion, which are hallmarks of aggressive tumors. Therefore, the aforementioned groups were used to analyze the association between tumor aggressiveness and CD97/CD55 expression. The tumors of the CD97/CD55^high^ group were more aggressive than tumors of the CD97/CD55^low^ group.

To further investigate the clinical importance of CD97 and CD55 expression in pancreatic cancer, the correlation between overall survival (OS) and CD97/CD55 expression was investigated. CD97/CD55^high^ expression was found to significantly correlate with a shorter OS. Kaplan-Meier plots demonstrated significantly poorer survival rates at all times points for the CD97/CD55^high^ group when compared with the CD97/CD55^low^ group (P<0.01; Fig. 3B). The overall three-year survival for all patients was 46%. However, the CD97/CD55^high^ group exhibited a significantly worse survival rate (16%) than the CD97/CD55^low^ group (63%). These results indicated that a progressive and significant deterioration in prognosis occurs with increased CD97/CD55 expression.

## Discussion

Pancreatic cancer is one of the most challenging types of cancer worldwide, and is a markedly aggressive disease with a poor overall prognosis and an incidence rate that approximately equals its mortality rate (3). Previous studies have indicated that adjuvant chemotherapy may improve survival in pancreatic cancer patients. Specific signaling pathways are already involved in therapeutic approaches or considered as potential therapeutic targets in pancreatic cancer (17,18). Recently, signaling pathways that mediate desmoplastic stromal response and tumor immunity have received attention as they may present promising therapeutic targets for future therapy (18). However, which patients would benefit from such therapy remains unclear.

In the present study, histological analysis of pancreatic cancer tissue specimens revealed a significant increase in CD97^+^/CD55^+^ epithelial cells, as percentage and intensity of expression were significantly higher in pancreatic cancer tissues when compared with that of adjacent normal pancreatic tissues. The involvement of CD97 in malignant tumor behavior may occur due to the interactions between CD97 and its receptor, CD55 (4). Recently, CD55 was identified as the ligand for CD97, which is a seven-transmembrane epidermal-like growth factor receptor that is involved in cell-cell and cell-matrix adhesion (6). In addition, previous studies have indicated that CD97 and CD55 are associated with malignancy and contribute to invasion and metastasis (8,19). Our results revealed that the tumor tissues with CD97/CD55^high^ expression were more aggressive than the tumor tissues exhibiting CD97/CD55^low^ expression. The upregulation of CD55 and the possible role of the CD55-CD97 interaction in invasion and metastasis promotion have led to the development of compounds which target CD55, as an immunotherapeutic approach for cancer treatment (14,20). Sutavani *et a*l (20) demonstrated that CD55 is a potent costimulator and activator of human naive CD4^+^ cells, resulting in the differentiation of a discrete Tr1 population that inhibits T-cell function in an IL10-dependent manner and maintains the Tr1 phenotype upon re-stimulation. CD97 and CD55 have been shown to be upregulated in a variety of solid tumors, including gastric and colorectal cancers (8,21–23). The expression levels of CD97 and CD55 in the abovementioned tumors were associated with the severity of the tumor, and were independent predictors of shorter OS in these patients.

CD97 and CD55 have been found in the tumor microenvironment and contribute to metastasis and invasion, leading to a poor prognosis (8). Although CD97 and CD55 are expressed by cells to protect them from the complement immune system, the presence of a small population that strongly expresses CD97 and CD55 predicts poor prognosis in a number of cancers. For example, Durrant *et al* (21) prospectively analyzed the correlation between CD55 expression and seven-year survival in 136 patients with colorectal cancer, and found that patients with tumors with high CD55 expression exhibited a significantly worse survival than patients with tumors exhibiting low CD55 expression. Furthermore, Wu *et al* (24) revealed that CD97 and CD55 are upregulated in human gallbladder carcinoma. The expression levels of CD97 and CD55 in gallbladder carcinoma were associated with tumor severity. In addition, levels of CD97 and CD55 expression were independent predictors of shorter OS in patients with gallbladder carcinoma (24). Mustafa *et al* (9) identified CD97 as a novel marker for dedifferentiated oral squamous cell carcinoma. The interaction between CD97 and CD55 may facilitate the adhesion of OSCC cells to the surrounding surfaces, which may lead to metastasis. The present study identified a significant correlation between the intensity of CD97 and CD55 expression and tumor aggressiveness and prognosis. A progressive and significant deterioration in prognosis as CD97 and CD55 expression increases has been shown.

In conclusion, the present study indicated that CD97 and its ligand CD55 are upregulated in pancreatic cancers and are closely associated with lymph node involvement, metastasis and vascular invasion. We propose that analysis of the expression of both CD97 and CD55 has a prognostic value for pancreatic cancer.
